# Cancer trends in Lebanon: a review of incidence rates for the period of 2003–2008 and projections until 2018

**DOI:** 10.1186/1478-7954-12-4

**Published:** 2014-03-04

**Authors:** Ali Shamseddine, Ahmad Saleh, Maya Charafeddine, Muhieddine Seoud, Deborah Mukherji, Sally Temraz, Abla Mehio Sibai

**Affiliations:** 1 Division of Hematology and Oncology, Department of Internal Medicine, American University of Beirut Medical Center, P.O Box 11–0236, Riad el Solh, Beirut 1107 2802, Lebanon; 2 Department of Obstetrics & Gynecology, American University of Beirut Medical Center, P.O Box 11–0236, Riad el Solh, Beirut 1107 2802, Lebanon; 3 Department of Epidemiology and Population Health, Faculty of Health Sciences, American University of Beirut, P.O Box 11–0236, Riad el Solh, Beirut 1107 2802, Lebanon

**Keywords:** Cancer, Lebanon, Surveillance, Trend analysis

## Abstract

**Background:**

The analysis of cancer incidence trends is essential to health care planning. The aim of this study is to examine variations in cancer incidence rates in Lebanon between 2003 and 2008 and use the observed trends to project cancer incidence until 2018.

**Methods:**

Using secondary data with a cumulative caseload of 45,753 patients from the National Cancer Registry database of the Ministry of Public Health in Lebanon, we estimated sex- and site- specific incidence of cancer for each year of the six-year period between 2003 and 2008. Logarithmic regressions were fitted to estimate the cancer incidence for the forecast years until 2018.

**Results:**

Between 2003 and 2008, males and females presented with an overall 4.5% and 5.4% annual increase, respectively. Significant increases were observed for cancers of the liver and prostate among males, and for cancers of the liver, thyroid, and corpus uteri among females. By 2018, incidence rates were projected to approach 296.0 and 339.5 cases per 100,000 for males and females, respectively. The most common five types of cancer are expected to be prostate, bladder, lung, non-Hodgkin, and colon among males; and breast, ovarian, non-Hodgkin, lung, and colon among females.

**Conclusion:**

The increased availability of screening programs and a growing smoking epidemic, most notably in women, are the most likely explanations behind the increased cancer incidence in the past decade. An aging population and higher proportion of older people suggest further increases in the cancer caseload in the future. The health care system in Lebanon will be required to adapt to the growing burden of cancer in our population.

## 

Cancer is the second leading cause of death after heart disease and stroke. Cancer also exerts the highest social and economic burden of all causes of morbidity through cost of diagnosis and treatment, and years of life lost due to disability [1]. Lebanon, a small middle-income country on the Eastern Mediterranean shore, is at the third stage of its demographic transition characterized by a decline in both fertility and mortality rates [2]. The United Nations estimates that the average life expectancy in Lebanon will rise from 71 to 78.7 years between 2009 and 2050, leading to an increase in the median population age from 28.8 to 41.7 years as well as the percentage of older population aged 60 years and above from 10.3% to 25.8% [3]. With an aging population, it is expected that cancer burden will increase and its associated economic toll will rise dramatically. The only study that examined cancer incidence at the national level dates back to 1998 and was based on data collated by the Lebanese Cancer Epidemiology Group (LCEG) [4]. In spite of the inherent limitations and challenges of the initial efforts, the study served as the first step toward a registry-based system and shaped the formulation of the National Cancer Registry in the country. Later on, several attempts at examining major sites of cancer in Lebanon and their epidemiological profile were made, but these have relied primarily on hospital-based tumor registries and hence were of limited generalizability. The present study examines variations in cancer incidence rates in Lebanon over a six-year period from 2003 to 2008 and uses observed trends to project cancer incidence until 2018. Findings from this study are crucial to anticipate future national health needs and develop accordingly strategies for disease prevention and control.

### Materials and methods

#### Six-year trend analysis from 2003 until 2008

The six consecutive years from 2003 until 2008 were drawn from published data as provided by the Lebanese National Cancer Registry database [5]. Using the *Joinpoint Regression Program (JRP),* sex-specific and age-standardized incidence rates for each year of study and each primary cancer site were calculated for the study period (2003–2008). Rates were standardized according to the World Standard Population.

The annual percent change (APC) in incidence rate was also calculated. The APC is based on the assumption that cancer rates change at a constant percentage of the previous year’s rate [6]. It allows comparing trends among scales for both rare and common diseases since it relies on percentages rather than increments. The criteria for characterizing the observed trends followed the NCI guidelines [7]: if the APC >0.5% with statistical significance, then the trend is judged to be rising; if the APC < −0.5% with statistical significance, then the trend is judged to be falling; if the −0.5% ≤ APC ≤ 0.5% with no statistical significance, then the trend is judged to be stable; and if the APC < −0.5 or APC >0.5 with no statistical significance, then the trend is judged to be a non-significant change (rise/fall). All the data that we analyzed were stratified by sex and presented as age-standardized rates (ASR). Statistical significance was also defined here at p-value ≤0.05.

Trend variations between the sexes were compared using two tests, parallelism and coincidence. Parallelism examines whether the regression mean functions (i.e., slope of the change in trend) are similar in direction between males and females, while coincidence indicates whether the regression mean functions are identical (i.e., similar overall incidence) between males and females [8].

#### Ten-year projected estimates from 2009 until 2018

Projections of the cancer incidence in Lebanon for the year 2018 were estimated using historical data covering the six-year period (2003–2008). The age-standardized rates from this period, the dependent variable, were fitted into a logarithmic model and the year(s) variable extending to 2018 as the independent variable in the model. The resulting rates from this model followed a logarithmic function over the study period. The logarithmic model was found to be best fitting and the most biologically reasonable model to describe the cancer incidence over time. Linear and log-linear regression models have been found to be the most practical means by which future patterns of cancer incidence can be estimated for periods of up to 10 to 15 years assuming no change in underlying trends [9]. These models assume a Poisson distribution for the observed number of incident cases, as predictions generated in this way are more reliable than those based on an assumption of normal distribution [10].

The fitted logarithmic model of the observed and imputed variables generated the new sex- and site-specific cancer incidence coefficients. The R square, which measures how well the model explains the total variations, and the p-value for the model were used to assess the goodness-of-fit of the model, considering a p-value of p ≤0.05 as significant.

### Results

#### Incidence trends (2003–2008)

The cumulative caseloads for the study period (2003–2008) included 45,753 patients, with males and females providing nearly equal and stable contribution across the years (48% and 52%, respectively). Table 1 provides the age-standardized rates among males and females for 2003 and 2008 separately and shows the APC over the six-year study period, which reflects the overall trend across the years and not necessarily the difference between 2003 and 2008, as two points in time.

**Table 1:** Sex-stratified (average) annual percent change (APC) by cancer site, 2003 until 2008 (per 100,000)

**Cancer site**	**Parameter**								**Comparison between genders**	
	**Males**				**Females**				**Parallelism**	**Coincidence**
	**2003**	**2008**	**APC**	**Trend**	**2003**	**2008**	**APC**	**Trend**		
Oropharyngeal	3.8	3.9	0.3%	Stable	2.6	2.6	5.0%	Rising	0.364	**<0.001**
Esophagus	--	0.9	−4.9%	Falling	--	0.5	8.4%	Rising	**<0.001**^**1**^	0.061
Stomach	6.2	8.1	2.9%	Rising	5.1	6.7	3.2%	Rising	0.839	**0.034**^**2**^
Colon	14.1	15.3	0.7%	Stable	13.1	14.1	−0.6%	Falling	0.964	0.069
Rectum	4.1	4.2	−1.0%	Falling	3.4	4.8	12.0%	Rising	0.060	0.124
Liver	1.8	4.0	13.6%	Rising*	1.5	3.9	18.3%	Rising*	0.492	**<0.001**^**2**^
Gall bladder	1.8	1.2	−5.1%	Falling	1.5	1.7	4.9%	Rising	0.680	**0.035**^**2**^
Pancreas	3.4	4.7	6.0%	Rising	2.9	2.7	−1.9%	Falling	0.217	0.320
Larynx	5.6	5.7	3.3%	Rising	1.3	1.6	3.6%	Rising	0.999	**0.020**^**2**^
Lung	31.6	31.8	0.3%	Stable	13.1	13.7	1.0%	Rising	0.433	0.479
Bone	1.4	2.2	5.18%	Rising	1.9	1.4	−3.0%	Falling	0.302	0.093
Connective tissue	5.3	3.3	−11.0%	Falling	2.8	2.7	−2.6%	Falling	0.167	0.240
Melanoma of the skin	3.04	2.1	−12.6%	Falling	1.5	1.9	−6.4%	Falling	0.503	0.124
Prostate	29.9	39.2	7.6%	Rising*						
Testis and male genitalia	4.3	3.3	−6.3%	Falling						
Breast					78.3	95.7	5.4%	Rising		
Cervix uteri					4.9	5.6	1.8%	Rising		
Corpus uteri					5.3	9.0	11.4%	Rising*		
Ovary					1.1	9.4	7.2%	Rising		
Bladder	29.5	34.0	3.3%	Rising	6.2	9.0	8.0%	Rising	0.116	**<0.001**^**2**^
Kidney and urinary tract	3.8	5.6	9.34%	Rising	2.2	2.4	1.0%	Rising	0.216	**0.014**^**2**^
Brain and nervous system	5.9	6.7	2.8%	Rising	3.3	5.0	5.2%	Rising	0.869	0.080
Thyroid	1.6	3.6	19.1%	Rising	4.2	8.2	12.7%	Rising*	0.153	**0.020**^**2**^
Hodgkin disease	4.2	4.1	0.25%	Rising	2.4	3.0	4.3%	Rising	0.354	**0.034**^**2**^
Non-Hodgkin disease	9.7	14.1	4.2%	Rising	7.5	11.6	5.9%	Rising	0.340	**<0.001**^**2**^
All sites	191.3	225.7	4.5%	Rising*	190.7	243.9	5.4%	Rising*	0.292	**0.020**^**2**^

APC values that are significant are indicated with an asterisk (*).

^1^A p-value ≤0.05 on the parallelism test indicates that the trends (slopes) between males and females are significantly different.

^2^A p-value ≤0.05 on the coincidence test indicate that the difference in the rates (position of trend lines) between males and females are significantly different.

#### Top cancer sites

Among males, age-standardized incidence rates for prostate cancer increased during the study period 2003–2008 from 29.9 to 39.2 cases per 100,000 (Table 1) and became the most-reported cancer site in 2008. This was followed by bladder cancer (ASR = 34.0 per 100,000) and lung cancer (ASR = 31.8 per 100,000), ranking second and third, respectively. Colon incidence rates ranked fourth and remained stable at 15.3 per 100,000 while non-Hodgkin lymphoma rates increased from 9.7 to 14.1 per 100,000 and became the fifth most-reported cancer site in 2008.

Among females, breast cancer remained the most-reported cancer site, with an increase in age-standardized incidence rate from 78.3 in 2003 to 95.7 cases per 100,000 in 2008 (Table 1). Colon cancer was the second most-common cancer at 14.1 cases per 100,000 despite a slight decrease over the six-year period. The third most common cancer was lung, increasing slightly to 13.7 cases per 100,000 in 2008. Although non-Hodgkin lymphoma was not among the 10 most-reported cancers in 2003, it rose in 2008 to the fourth most-reported malignancy. Ovarian cancer ranked as the fifth most-common cancer in 2008 with 9.4 cases per 100,000.

#### Annual percent change (APC)

Among males, three out of the 21 considered cancer sites showed a stable trend across the study period (2003–2008), six showed a decreasing trend, and 12 showed an increasing trend, statistically significant in the case of liver cancer and prostate cancer (APC of 13.6% and 7.6% respectively). Among females, out of the 23 considered sites, five sites showed a decreasing trend, and 18 sites showed an increasing trend, statistically significant for liver cancer, thyroid cancer, and corpus uteri (APC of 18.3%, 12.7%, and 11.4%, respectively). Overall, males presented with a 4.5% (*P < 0.05*) annual increase while females presented with 5.4% (*P <0.05*) annual increase over the study period (Table 1).

#### Comparison of trends between genders

In contrast to males, females showed a steep annual increase in esophageal cancer between 2003 and 2008 (*P <0.001*), but this resulted only in a marginal difference on their trend coincidence (*P <0.061*). Cancers of the rectum, gall bladder, pancreas, and bone had opposing trends in the direction of change across gender, and this was most notable for cancer of the rectum (*P <0.06*). For cancers of parallel direction of change, females had a significantly higher annual trend change for cancers of the liver, bladder and Hodgkin disease than males; while males had a significantly higher annual trend change for cancers of the kidney and urinary tract and thyroid than females (Table 1).

#### Projected cancer rates (2009–2018)

Table 2 presents the projected incidence rates for the year 2018. Overall, cancer incidence rates in 2018 are expected to approach 296.0 and 339.5 cases per 100,000 for males and females, respectively. The three most common types of cancer in males in 2018 are expected to be prostate, bladder, and lung, accounting for approximately half of all cancer cases. The most commonly diagnosed cancers in females in 2018 are expected to be cancers of the breast, ovarian, and non-Hodgkin disease, accounting for approximately half of all cancers. The R squares show that the best-fitted models (R^2^ ≥ 0.8) for males were for prostate (0.81), while the best-predicting models in females were thyroid and corpus uteri (0.92 and 0.91, respectively).

**Table 2:** Projected incidence rates for cancer of major sites for the year 2018 (per 100,000)

**Cancer site**	**Males**		**Females**	
	**Projected incidence rates**	**(R**^**2**^**)**	**Projected incidence rates**	**(R**^**2**^**)**
	**2018**		**2018**	
Oropharyngeal	3.7	0.03	2.8	0.04
Esophagus	0.6	0.15	0.7	0.25
Stomach	9.3	0.21	7.7	0.08
Colon	17.1	0.26	10.9	0.03
Rectum	4.4	0.50	5.9	0.32
Liver	7.9	0.79*	6.6	0.62
Gall bladder	0.7	0.13	2.5	0.23
Pancreas	5.9	0.33	2.3	0.08
Larynx	7.9	0.26	2.0	0.29
Lung	32.7	0.04	14.3	0.07
Bone	3.8	0.19	1.1	0.15
Connective tissue	0.4	0.07	1.6	0.03
Melanoma of the skin	0.3	0.35	1.0	<0.01
Prostate	64.2	0.81*		
Testis and male genitalia	0.5	0.37		
Breast			137.0	0.64*
Cervix uteri			5.8	0.09
Corpus uteri			16.0	0.91*
Ovary			22.0	0.49*
Bladder	41.2	0.29	13.4	0.64
Kidney and urinary tract	9.4	0.65	2.2	0.02
Brain and nervous system	7.1	0.08	1.6	0.18
Thyroid	7.0	0.72*	14.7	0.92*
Hodgkin disease	4.3	0.01	4.9	0.24
Non-Hodgkin disease	22.1	0.34	20.0	0.39
All sites	296.0	0.67*	339.5	0.75*

R^2^(R-square) values that are significant with a p-value ≤0.05 are noted with an asterisk (*), indicating a significantly fit model.

### Discussion

Early incidence rates for cancer in Lebanon were provided by Abou-Daoud in 1966. Cancer rates were reported at 102.8 and 104.1 per 100,000 for males and females, respectively [11]. The study observed high incidence of lung, bronchus, and larynx cancer among males, while females presented with high incidence of breast and cervix uteri cancers. Thirty years later, a national study conducted by the Lebanese Cancer Epidemiology Group in collaboration with all hospitals, oncology specialists, and pathology centers in the country included a total of 4,388 incident cases and reported national age-standardized incidence rates of 154.2 and 134.8 per 100,000 for males and females, respectively [4]. For males, bladder cancer was the most commonly reported cancer followed by cancers of the prostate and the lung. For females, breast cancer remained the most common cancer and accounted for one-third of all reported cases. A national cancer registry was then established under the auspices of the Ministry of Public Health and published its first annual report on national cancer incidence data in 2003, noting age-standardized incidence rates of 191.3 and 190.7 per 100,000 for males and females, respectively [5]. Bladder, prostate, and lung cancers remained the highest-reported malignancies among males. For females, breast cancer remained the highest-reported cancer, followed by colon cancer and non-Hodgkin lymphomas.

These major landmarks in the surveillance of cancer in Lebanon show an increase in the incidence rates across time and a change in the epidemiology of the disease. Figures 1, 2, 3 and 4 reproduce the data generated from this study and present incidence rates for the five most common cancers in males and females across the years of the study (2003–2018). Cancers of prostate, bladder, lung, colon, and non-Hodgkin were the most common among males, and cancers of the breast, colon, lung, non-Hodgkin, and ovary were the most common among females. Currently, Lebanon includes the highest proportion of older people in the Arab world (10% for those aged 60 years and over) [2]. Whilst aging remains the most compelling explanation for the rise in cancer rates over the study period from 2009 until 2018, we also note a disproportionate rise in smoking-related cancers represented by malignancies of the lungs, trachea, and larynx. Females still lag behind males in the incidence of such cancers, but they are following a similar incremental increase. Bladder cancer has been singled out as a cancer with an unusually high incidence, with a pattern that exceeds other regional and international estimates [12].

**Figure 1:**
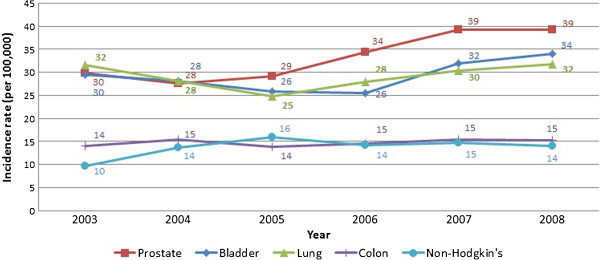
Cancer incidence of the five most common cancers in males (2003-2008).

**Figure 2:**
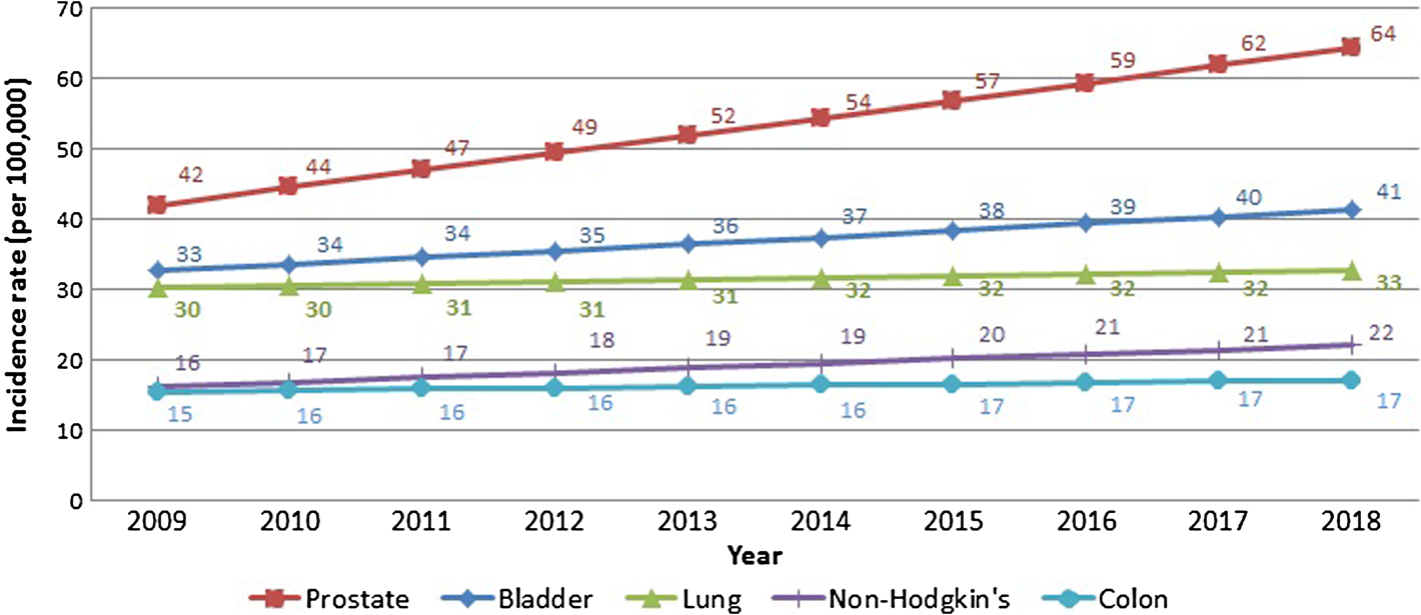
10-year cancer incidence projection of the five most common in males (2009-2018).

**Figure 3:**
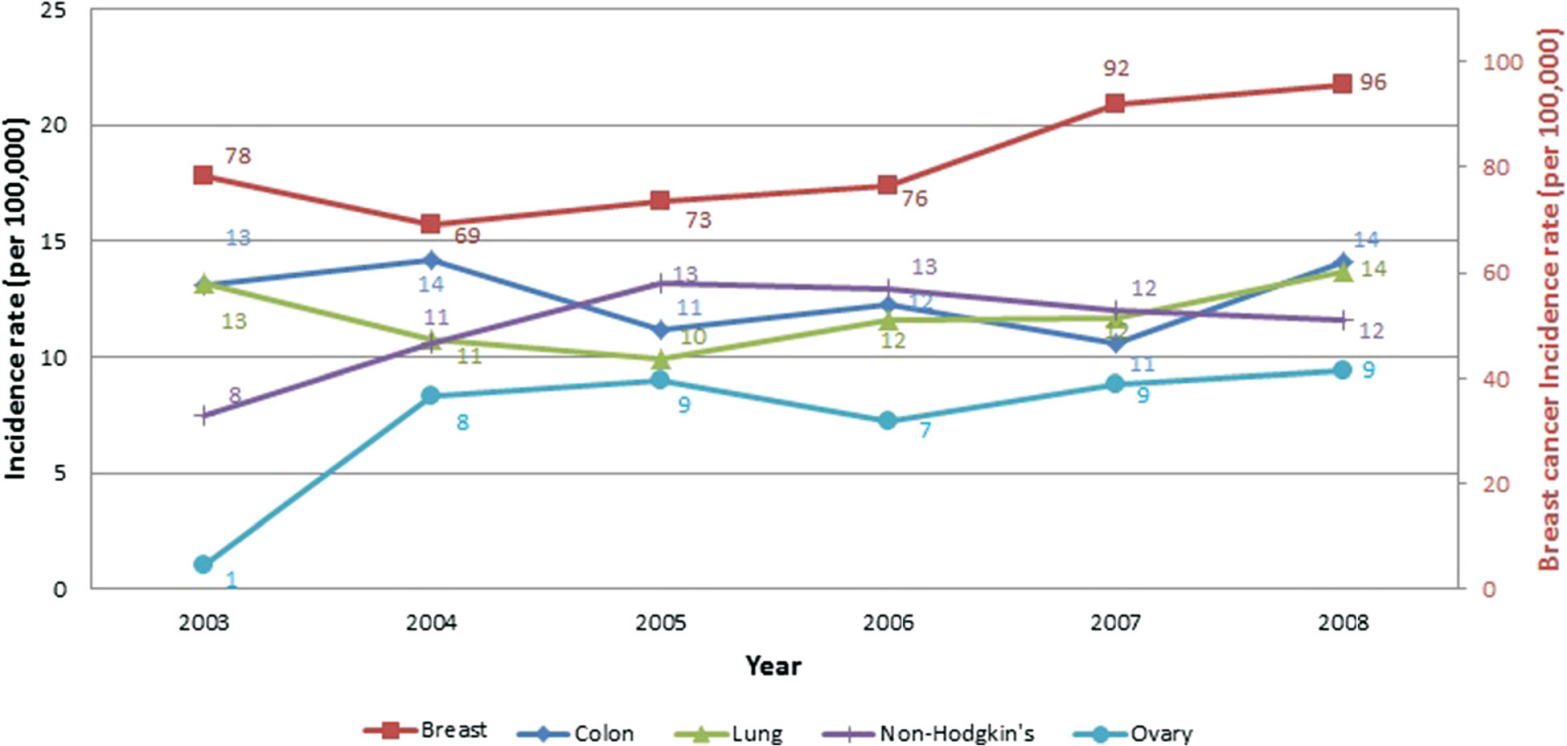
Cancer incidence of the five most common cancers in females (2003-2008).

**Figure 4:**
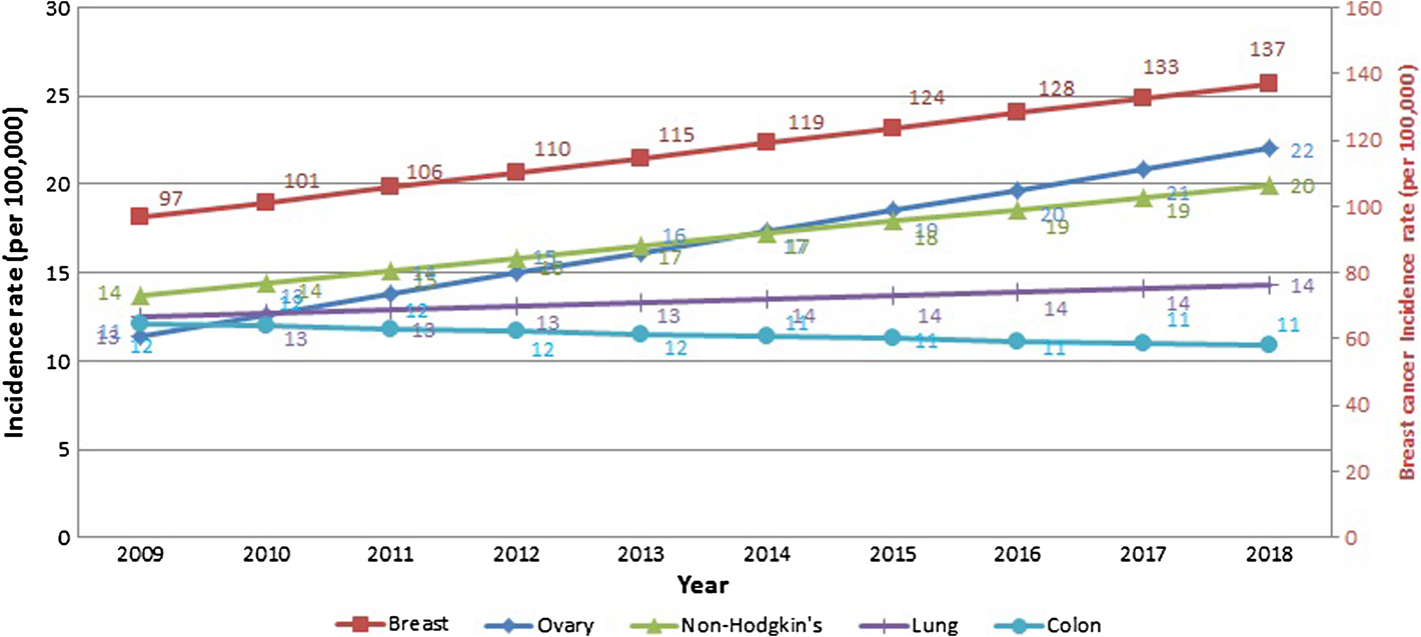
10-year cancer incidence projection of the five most common cancers in females (2009-2018).

Prostate cancer follows as the third most-reported cancer in men. Prostate-related cases aggregate in those 65 years and above, showing an older age at diagnosis [5]. The increased utilization of PSA screening may partially account for some of this increasing trend; however, projections indicate that this trend in prostate cancer is expected to be maintained until 2018. Similarly, the high occurrence of breast cancer among women may also be related to increased use of mammographic screening and early detection, an effort assisted by major awareness campaigns that started in 2004.

#### Change in the contribution of all cancers

Between 2003 and 2008, females had a faster expansion of the contribution of the most common cancer sites compared to males. Between 2008 and 2018, this differential is reduced as the contribution of the common cancer sites seems to expand at similar pace. We relate this to differentials in the maturity of the cancer epidemic between females and males. Males are approaching the saturation of their risk factors and hence the incidence of cancer cases, with the corresponding trend becoming stable for lung cancer and decreasing for bladder cancer. Females are increasingly being exposed to more risk factors than before, with maturation of the smoking epidemic and an increase in the incidence of smoking-related cancers.

An increasing trend was observed in most of the cancer sites in the period from 2003 to 2008 (Table 1). However, a few cancers showed a non-significant decreasing trend mainly in esophageal and melanoma of the skin. The rates in these sites were initially low, which makes any variation in these rates appear large when compared to the initial estimates. The decrease in connective tissue cancer rate was attributed to the adoption of a more specific classification by topography than an actual decrease.

#### Change in the ranking of cancer sites

Among males, lung cancer was the most common cancer in 2003 with 31.6 cases per 100,000 cases. This was followed by prostate, bladder, and colon cancer. In 2008, significant increases in prostate cancer made it to the top of the list with 39.2 cases per 100,000 cases. Similarly, bladder cancer rose to become the second most-common reported cancer site among males with 34.0 cases per 100,000 cases, and lung cancer became the third most-common type. Such a ranking is projected to be maintained until 2018.

Between 2003 and 2008, the most notable changes in females were the rise in breast cancer incidence and the appearance of ovarian cancer as the fifth most commonly reported cancer site. In 2018, breast cancer is projected to remain the most-common reported cancer site, while ovarian cancer is projected to increase and rank as the second most-common reported site. This will be followed by non-Hodgkin disease with 20.0 cases per 100,000. A recent nationwide lymphoma study in Lebanon noted an increase in lymphomas worldwide over the past decade and argued for the need of large scale studies to assess the increasing rates [13]. Worldwide, an annual increase of 1-2% in non-Hodgkin lymphoma incidence has been noted, mostly in older people aged over 55 years [14].

#### Smoking-related cancers

Almost all smoking-related cancers in both sexes showed a rising trend. Lung cancer showed a steeper rise in females compared to males. Tobacco smoking in Lebanon is a major health concern; marketing of tobacco products is permissible and smoking is an acceptable social habit. Additionally, narguile smoking (also known as Hubble-Bubble or Hooka) with health effects similar to that of cigarette smoking is increasingly becoming more prevalent in this population. Adult smoking is estimated at 38.5% (males at 46% and females at 31%), and smoking among youth shows much higher estimates (65.8% for boys and 54.1% for girls) [15]. Rates of smoking in females are considered the highest in the region and are rising at a higher pace than those observed in neighboring countries [16-18]. This proliferation of tobacco products and smoking habits may explain the continuing rise in such smoking-related cancers as lung and bladder cancers, in both males and females.

#### Breast cancer

In Lebanon, breast cancer in females presents at a younger age, with a median of 50 years, compared to western countries where the median age approaches 63 years [19]. Moreover, the incidence of breast cancer among women in general is increasing (APC = 5.4%) and is projected to continue to cause the highest morbidity burden compared to other cancers (33%-44%). These trends can be partly explained by widespread national breast cancer awareness campaigns since the early 2000s, most notably in 2003–2004, as well as the wide adoption of mammography screening programs at reduced fees within NGOs and primary health care centers. Awareness campaigns increase women’s personal motivation to utilize the diagnostic services and sensitize physicians to suspect and diagnose signs and symptoms of potential breast tumors [19]. Further, significant changes in marriage and fertility trends, with higher ages of marriage and fewer desired children, are likely to increase the likelihood of breast cancer incidence in the country [20]. Breast cancer is also altered by changes in dietary habits. The nutritional transition, characterized by an alteration in the food choices from the traditional Mediterranean diet to a more Westernized diet is currently underway [21,22]. National studies from Lebanon [23,24] report increasing rates of overweight and obesity that is more evident in females and consistent with the growing epidemic of obesity worldwide.

#### Bladder cancer

We continue to observe a high incidence of bladder cancer relative to other cancers among males and females, and this trend remained stable throughout the study period. Although the incidence of bladder cancer has been related to exposure to chlorinated water and its byproducts and caffeine consumption [25,26], smoking remains one of the most direct risk factors [27]. Research on the higher incidence of urinary bladder cancer in the United States and Argentina relative to Europe and worldwide has been related to the type of curing of the cigarettes used in these countries [28]. In particular, black tobacco (air-cured) has shown to be associated with higher rates of urinary bladder cancer than blond tobacco (flue-cured) [29]. In Lebanon, although the imported cigarettes alternate between black and blond cigarettes, the majority of the smoking population uses black tobacco while smoking narguile. The tobacco used for narguile smoking is locally grown and later air-cured (oriental tobacco), and this may explain the high rates in urinary bladder cancer in the country compared to worldwide and regional estimates.

#### Strengths and limitations

This study makes use of all the available six-year data to study the direction and magnitude of the trend and project cancer cases and incidence until 2018. The projected estimates in this study, however, should be examined with caution since projections assume constant incidence and trend changes. Projected estimates can be either an underestimate due to rise in known and unknown risk factors or an overestimate due to enhanced primary preventive efforts that might result in decrease of cancer incidence rates. While current lifestyle-related risk factors are not necessarily associated with increased cancer rates due to the relatively short latency period, an aging population and increasingly higher proportion of elderly with higher life expectancy are consistent with increase in the burden of cancer in the future.

### Conclusion

Smoking-related cancers continue to be fueled by changes in lifestyle and the growing smoking epidemic in Lebanon, most notably in women. Moreover, increase in life expectancies and a higher proportion of older people will further inflate cancer burden and overall incidence rates. The health care system in Lebanon will be required to adapt to the increasing burden of cancer in our population. The limited resources, however, call for prioritizing prevention and control focusing on smoking-related cancer and continue promoting early breast screening programs. Studies are needed to examine reasons behind the notably high incidence of bladder cancer in Lebanon.

### Competing interests

The authors declare that they have no competing interests.

### Authors’ contributions

ASh conceived of the study and participated in its design and interpretation of the data. AMS participated substantially in the interpretation of the data and reviewed and assisted in the statistical analysis and the drafting and editing of the paper. ASa participated in the design, carried out the statistical analysis, and drafted the paper. MC carried out statistical analysis and participated in drafting and editing the paper. MS, DM, and ST assisted in the interpretation of the data and drafting of the paper. All authors have read and approved the final manuscript.
